# A candidate probiotic with unfavourable effects in subjects with irritable bowel syndrome: a randomised controlled trial

**DOI:** 10.1186/1471-230X-10-16

**Published:** 2010-02-10

**Authors:** Solveig C Ligaarden, Lars Axelsson, Kristine Naterstad, Stian Lydersen, Per G Farup

**Affiliations:** 1 Department of Medicine, Innlandet Hospital Trust, Gjøvik, Norway; 2 Unit for Applied Clinical Research, Department of Cancer Research and Molecular Medicine, Norwegian University of Science and Technology, Trondheim, Norway; 3 Nofima Mat AS, Ås, Norway

## Abstract

**Background:**

Some probiotics have shown efficacy for patients with irritable bowel syndrome (IBS). *Lactobacillus (L.) plantarum *MF1298 was found to have the best *in vitro *probiotic properties of 22 strains of lactobacilli. The aim of this study was to investigate the symptomatic effect of *L. plantarum *MF1298 in subjects with IBS. Primary outcome was treatment preference and secondary outcomes were number of weeks with satisfactory relief of symptoms and IBS sum score.

**Methods:**

The design was a randomised double blind placebo-controlled crossover trial. 16 subjects with IBS underwent two three-week periods of daily intake of one capsule of 10^10 ^CFU *L. plantarum *MF 1298 or placebo separated by a four-week washout period.

**Results:**

Thirteen participants (81%; 95% CI 57% to 93%; *P *= 0.012) preferred placebo to *L. plantarum *MF1298 treatment. The mean (SD) number of weeks with satisfactory relief of symptoms in the periods with *L. plantarum *MF1298 and placebo were 0.50 (0.89) and 1.44 (1.26), respectively (*P *= 0.006). IBS sum score was 6.44 (1.81) in the period with *L. plantarum *MF1298 treatment compared with 5.35 (1.77) in the period with placebo (*P *= 0.010). With a clinically significant difference in the IBS sum score of 2 in disfavour of active treatment, the number needed to harm was 3.7, 95% CI 2.3 to 10.9.

**Conclusions:**

This trial shows for the first time an unfavourable effect on symptoms in subjects with IBS after intake of a potential probiotic.

**The trial registration number:**

Clinical trials NCT00355810.

## Background

Irritable bowel syndrome (IBS) is the most frequent functional gastrointestinal disorder, with a prevalence of 5-11% in most countries [1]. The workload generated by IBS is considerable and constitutes approximately one-third of all visits to gastroenterologists [2]. It is a biopsychosocial disorder that requires a multifactorial approach [3]. No proper treatment is available.

The human gut contains over 1000 different bacterial species and an indeterminate number of strains of which a minority of the strains is cultivable [4] Probiotics are defined as "live microorganisms which when administered in adequate amounts confer a health benefit on the host" [5]. Some studies have shown beneficial effect of probiotics in IBS [6]. *Lactobacillus *(*L*.) *plantarum *299v reduced flatulence and abdominal pain in patients with IBS [7]. *L. plantarum *MF1298 was found to have the best *in vitro *probiotic properties of 22 strains of lactobacilli isolated from fermented food products. This strain was confirmed to adhere to the human colon adenoma cell line CaCo2, to strengthen transepithelial resistance of a CaCo2 cell layer and to increase production of certain tight junction proteins, to have antimicrobial activity against potential pathogens, and to survive passage through the human gastrointestinal tract [8-10]. *L. plantarum *MF1298 was therefore proposed as a potential candidate probiotic strain.

The aims of this randomised placebo-controlled crossover trial were to study the effect of *L. plantarum *MF1298 on treatment preference, satisfactory relief of symptoms and symptoms in subjects with IBS.

## Methods

### Participants

Participants were recruited from a hospital-based gastroenterology outpatient clinic and a private gastroenterological practice. Subjects 18 to 75 years of age with IBS according to the Roma II criteria and symptoms the last three months were eligible for inclusion. All subjects had had a sigmoidoscopy or colonoscopy performed within the last five years to exclude organic disease. Other tests to confirm the diagnosis were performed at the physicians' discretion. Pregnant and breast-feeding women and subjects with major psychiatric, mental or behavioural disorders, coexisting gastrointestinal and other disorders that might influence the symptoms, or poor knowledge of language were excluded, as were those who had used probiotics more than once a week in the previous three weeks or antibiotics or laxatives in the previous five weeks. The study was made in accordance with the Helsinki Declaration and all participants gave written informed consent to participation before enrolment. The Regional medical research ethics committee, Central Norway approved the study protocol.

### Study design

The study was a randomised double blind, placebo-controlled, crossover trial with a one-week run-in period followed by randomisation and two three-week treatment periods separated by a four-week washout period. Participants with satisfactory relief of symptoms in the run-in period were excluded from further participation. IBS symptoms were recorded on diary cards every evening during the run-in period, during the last week of the washout period, and the last week of the two treatment periods. Satisfactory relief of symptoms was recorded on diary cards at the end of the run-in and washout periods and at the end of each week during the treatment periods. At the end of the study, the participants recorded treatment preference for one of the treatment periods. Faecal samples were collected at the end of the run-in, washout period, and the two treatment periods. All data were collected at the hospital based gastroenterology outpatient clinic at Innlandet Hospital Trust, Gjøvik.

The computer-based randomisation was performed at the Unit for Applied Clinical Research, Norwegian University of Science and Technology, Trondheim, Norway. Faun Pharma, Norway, provided packed and numbered containers with the capsules containing 10^10 ^CFU live, freeze-dried *L. plantarum *MF 1298 or placebo according to the randomisation list. The capsules were confirmed to contain the correct number of pure *L. plantarum *MF1298 by classical and genetic methods, and were checked for the presence of common pathogens. The capsules looked identical and were prescribed to be taken once daily with liquid. The participants and health care providers were blinded until data entry was complete.

### Assessments

The participants were asked about treatment preference (the period with least symptoms) at the last visit, and about satisfactory relief of symptoms (yes/no) at the end of the run-in and washout periods and at the end of each week during the treatment periods. Seven gastrointestinal symptoms were recorded. Abdominal pain/discomfort, urgency and bloating were recorded as none, mild, moderate, or severe (score 0-3); stool frequency as number of stools per day; stool consistency according to Bristol stool scale form (score 1-7); and straining and incomplete bowel movement as yes/no (score: 1 or 0) [11]. An IBS sum score (score 0-15) was calculated as the sum of these seven scores after "normalisation" of stool frequency and consistency to achieve low scores for normal bowel habits. The "normalisation" was performed as follows: Stool frequency: 0 stool/day = 1; 1-3 stools/day = 0; 4-5 stools/day = 1; ≥ 6 stools/day = 2. Stool consistency: Bristol stool scale 3-5 = 0; Bristol stool scale 2 and 6 = 1; Bristol stool scale 1 and 7 = 2. A diarrhoea score was calculated as the sum of the "none normalised" scores of stool frequency and stool consistency. Assessment of compliance was based on returned capsules.

Faecal samples, frozen in Carey Blair medium (Oxoid Ltd, Basingstoke, Hampshire, UK), were analyzed for detection of *L. plantarum *by real-time PCR using 50 cycles. Primers: 5'-TGG ACC GCA TGG TCC GAG-3' (F) and 5'-GTG AGC CGT TAC CCC ACC AT-3' (R), and the Taqman probe 5'-TCC CGC GGC GTA TTA-3', targeting a specific *L. plantarum *region of the conserved 16S rDNA sequence, were used in the analysis. Verification of specificity and control of efficiency of the primer-probe pair were performed according to standard procedures, and will be documented elsewhere (Rudi et al., manuscript in preparation).

### Outcomes

The primary outcome measure was treatment preference. Secondary outcomes were the number of weeks with satisfactory relief of symptoms and the IBS sum score. All comparisons were between treatment with *L. plantarum *MF1298 and placebo. Presence of *L. plantarum *MF1298 was assessed by analysis of faeces as described above. Adverse events were noted.

### Statistical methods

The sample size calculation was based on the treatment preference (the proportion of participants preferring one treatment period to the other). Nineteen participants were required to reveal a treatment preference of 80% compared with the null hypothesis value of 50%, with 80% power at a two-sided significance level of 5%.

Changes within and between groups were compared with paired *t *test and independent *t *test, respectively. The confidence interval and *P *value for treatment preference were calculated with the Wilson (score) method. The difference between treatment periods as regards number of subjects with satisfactory relief of symptoms for zero, one, two and three weeks was tested with the marginal homogeneity test for matched ordinal variables. The number needed to harm was calculated by the method described by Walter, with a difference ≥ 2 in the IBS sum score between *L. plantarum *MF1298 and placebo regarded as clinically significant [12]. Two-sided *P *values < 0.05 were considered statistically significant, and the 95% confidence interval (CI) was calculated for the main outcomes. Modified intention-to-treat analysis was performed. All results are given as mean (SD) unless otherwise indicated.

## Results

**Figure 1:**
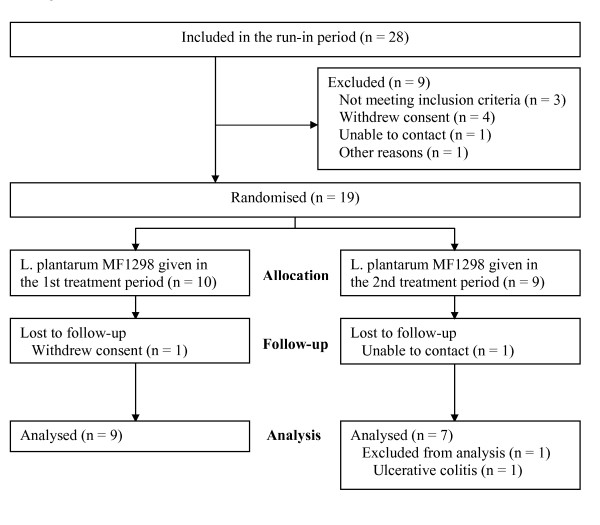
**Flow chart of the participants through the trial**.

Twenty-eight participants were included between January and April 2006. Figure 1 shows the flow of participants through the trial. Sixteen participants (five males and eleven females) with a mean age of 50 (11) years and BMI 24 (3) kg/m^2 ^were available for the modified intension-to-treat analysis; one had constipation-predominant, nine alternating, and six diarrhoea-predominant IBS. The IBS sum score at run-in was 6.21 (1.63), and the duration of symptoms was 31 (17) years. Four participants with protocol violations were included in the modified intention-to-treat analyses; two had inadequately completed diary cards (three and five days, respectively), one used an antibiotic during the active period, and one received supplementary treatment for IBS in the placebo period.

**Figure 2:**
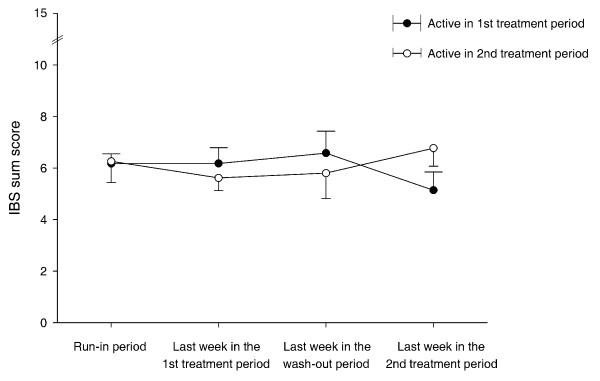
**IBS sum score during the trial by allocation group**. The results are given as mean with SEM.

Thirteen participants (81%; CI 57% to 93%; *P *= 0.012) preferred placebo to *L. plantarum *MF1298 treatment. The number of weeks with satisfactory relief of symptoms was statistically significantly higher in the placebo period compared with the *L. plantarum *MF1298 period (Table 1 and 2) and the IBS sum score and the score for diarrhoea were significantly higher in the period of *L. plantarum *MF1298 treatment than with placebo (Table1). The subclasses of IBS (diarrhoea predominant, constipation predominant, and alternating) showed the same tendency for higher IBS sum scores in the period with *L. plantarum *MF1298 compared with placebo (data not shown). Figure 2 shows the IBS sum scores during the trial by allocation group. The IBS sum score in the active period was 6.44 (1.81), in the placebo period 5.35 (1.77), and the correlation between these was 0.66. The difference in IBS sum score between active treatment and placebo was 1.09 (1.47). The resulting proportion of subjects with a score difference of at least 2 in disfavour of active treatment was 27% (CI 9% to 44%), and the number needed to harm was 3.7 (CI 2.3 to 10.9).

**Table 1:** Daily symptom scores and number of weeks with satisfactory relief of symptoms during the two treatment periods.

Symptoms	LpMF1298	Placebo	Paired differences, mean (CI)	Statistics
Number of weeks with satisfactory relief of symptoms	0.50 (0.89)	1.44 (1.26)	-0.94 (-1.57 to -0.31)	*P *= 0.006

Individual symptoms				
Abdominal Pain/Discomfort	1.55 (0.57)	1.14 (0.55)	0.41 (0.09 to 0.73)	*P *= 0.016
Stool frequency (normalised)	0.15 (0.18)	0.19 (0.21)	-0.03 (-0.14 to 0.07)	*P *= 0.48
Stool consistency (normalised)	0.86 (0.55)	0.61 (0.55)	0.25 (-0.12 to 0.61)	*P *= 0.17
Urgency	1.54 (0.59)	1.12 (0.56)	0.42 (0.17 to 0.66)	*P *= 0.002
Bloating	1.23 (0.59)	1.16 (0.68)	0.07 (-0.31 to 0.46)	*P *= 0.69
Straining	0.51 (0.37)	0.58 (0.40)	-0.07 (-0.17 to 0.03)	*P *= 0.13
Incomplete bowel movement	0.59 (0.35)	0.54 (0.41)	0.05 (-0.06 to 0.17)	*P *= 0.35
Sum symptoms				
IBS sum score	6.44 (1.81)	5.35 (1.77)	1.09 (0.31 to 1.87)	*P *= 0.010
Stool characteristics				
Stool frequency	1.52 (0.68)	1.33 (0.58)	0.19 (-0.07 to 0.45)	*P *= 0.15
Stool consistency	4.84 (1.51)	4.17 (1.31)	0.67 (0.20 to 1.13)	*P *= 0.008
Diarrhoea (consistency + frequency)	6.36 (1.99)	5.50 (1.71)	0.86 (0.33 to 1.39)	*P *= 0.004

The results are given as mean(SD).

**Table 2:** Number of subjects with satisfactory relief of symptoms for 0, 1, 2, and 3 weeks in the two treatment periods.

			Number of subjects with satisfactory relief of symptoms for 0, 1, 2, and 3 weeks in the placebo period				Total no. of subjects
			
			0 week	1 week	2 weeks	3 weeks	
Number of subjects		0 week	4	4	1	2	11
with satisfactory relief		1 week	1	0	0	2	3
of symptoms for 0, 1, 2, and 3 weeks		2 weeks	0	0	1	0	1
in the LpMF1298 period		3 weeks	0	0	0	1	1

Total no. of subjects			5	4	2	5	16

The difference in favor of placebo was statistically significant (*P *= 0.012).

*L. plantarum *was not detected in the faeces in any of the subjects in the run-in period, in the washout period (except for in one subject given active treatment in the first period), nor in the placebo period. However, L. *plantarum *was detected in all faecal samples at the end of the active treatment period, indicating that the analysis was targeting strain MF1298.

Compliance with intake of drugs was 95%. Two participants did not return their unused drugs after the last treatment period.

One participant had a short stay in hospital for cervicobrachialgia during the washout period, two weeks after the end of active treatment. There was no organic explanation and she continued in the trial. Three minor adverse events were noted.

## Discussion

The study shows an unfavourable effect on symptoms in subjects with IBS after intake of *L. plantarum *MF1298 compared to placebo. To our knowledge, similar unfavourable effects of probiotics have not been reported in subjects with IBS. Other studies with probiotics in subjects with IBS show either no effect or a favourable effect [6]. The divergent results could be related to different probiotic properties and health effects of the genera, strains, and species in use. Quigley assumed the possible superiority of *Bifidobacterium *spp for treatment in IBS [13]. *Bifidobacterium *(*B*.) *animalis *DN-173010 increased stool frequency in subjects with constipation at entry in a large study [14]. O'Mahony et al. compared the symptomatic effect of *L. salivarius *UCC4331 and *B. infantis *35624 in subjects with IBS [15]. *B. infantis *35624 induced a favourable effect on IBS symptoms. In a second study by the same researchers, the beneficial effect of *B. infantis *35624 was confirmed [16]. Lactobacilli have been evaluated in several trials with inconsistent results, but no deleterious effects have been reported. One trial of *L. reuteri *ATCC 55730 showed no significant effect on gastrointestinal symptoms in patients with IBS, while another trial also in patients with IBS showed no effect of *L. casei *strain GG [17]. *L. casei *strain GG in combination with other probiotics showed a positive effect on IBS symptoms in one study by Kajander [18]. Two trials with *L. plantarum *299v showed a reduction of abdominal pain and flatulence, while a small crossover study found no effect on symptoms of IBS [19].

Reports of unfavourable effects of probiotics are rare and probiotics have until recently been regarded as safe [6]. Untoward effects were reported in only three out of 185 human studies [20]. A strain of *L. acidophilus *increased faecal protein catabolites in healthy volunteers in one study, while *Saccharomyces cerevisiae *increased disease activity in patients with stable Crohn's disease in one study, and in another study increased serum glucose in healthy volunteers [20]. Sepsis has been reported in some subjects using probiotics [21]. The most alarming report was published in 2008, showing increased mortality of severe acute pancreatitis following treatment with a multispecies probiotic preparation [22].

The doses of probiotics used for the treatment of IBS in other trials vary from 2 × 10^8 ^to 2 × 10^10 ^CFU per day [7,15,19,23,24]. In a dose-finding study, the optimal dose of *B. infantis *35624 was 1 × 10^8 ^CFU which was superior to placebo, 1 × 10^6 ^CFU and 1 × 10^10^ CFU. However, the 1 × 10^10 ^CFU dose was associated with significant formulation problems [16]. The only previous study in humans with *L. plantarum *MF1298 is a study of the survival and persistence of the strain in the gastrointestinal tract in 17 healthy volunteers. They were given 6 × 10^9 ^CFU per day of *L. plantarum *MF1298 either as a freeze-dried preparation or present in 15 g fermented sausage. No gastrointestinal symptoms or other adverse events were spontaneously reported, but such symptoms were not systematically recorded [10]. The dose of 1 × 10^10 ^CFU *L. plantarum *MF1298 selected for this study was in the same order as the doses used in other studies with lactobacilli [20]. Despite the lack of evidence for a reduced or detrimental effect of high doses, we cannot exclude that a too high dose might have contributed to the unfavourable outcome of this study.

It has been proposed that the most potent probiotics may have increased pathogenicity [21]. The multispecies probiotic preparation used in the study of acute, severe pancreatitis where mortality was increased, was composed of six strains [22]. These strains, selected from 69 different probiotic bacteria, had better probiotic properties in combination than the individual components. *L. plantarum *MF1298 had the best *in vitro *probiotic properties of 22 strains [8-10]. The probiotic with the best probiotic properties as determined *in vitro *is not necessarily the best one to "confer a health benefit on the host".

The possibility of contamination of capsules by pathogens was excluded in our study, but the presence of endotoxins in the *L. plantarum *MF1298 and placebo preparations was not checked. This is, however, unlikely to be the reason for the unfavourable effect, because the company providing the capsules is a reliable producer of food supplements.

The serious adverse event and three minor adverse events reported were judged to be unrelated to the treatment.

### Strengths and weaknesses

Probably due to the heterogeneity of the sample in terms of bowel habit predominance, we cannot point to aggravation of a specific symptom. But all outcomes, both the primary outcome (treatment preference) and the secondary outcomes (number of weeks with satisfactory relief of symptoms and IBS sum score), show the same unfavourable direction for active treatment. This strengthens the internal validity, but a type I error cannot be excluded.

With an IBS sum score difference of 2 chosen as clinically significant, the number needed to harm was 3.7. A 2-point difference on a scale with a range of 15 means 13%, and a change of 10% is often regarded as significant on such scales. However, the IBS sum score was not validated for responsiveness and clinically significant differences. Considering that the mean IBS sum score in the run-in period was 5.97, the score difference of 2 chosen as clinically significant might be rather high.

In this study the mean age of subjects was 50 years and the proportions of subjects with diarrhoea predominant, constipation predominant, and alternating IBS were 38%, 6%, and 56%, respectively. In corresponding studies the participants were younger and the proportions of subjects in the subgroups were more balanced [1,25]. The older age and the somewhat different distribution of subgroups in our study raise the question of external validity. A beneficial effect in younger subjects, in subjects with more or less symptoms compared with our participants, in subgroups of subjects (such as constipation predominant), or in populations with other dietary habits and gut microflora cannot be excluded. Furthermore, a longer period of intervention would have strengthened the internal validity, but increased the drop-out rates.

The advantage of the crossover design used in this study is the increase in power of within-participant comparisons, and thus its requirement for fewer participants. For ethical reasons, the number of participants and the study period should be reduced as much as possible in a phase II study like this one. The design is fitted for stable chronic diseases [26]. Although IBS is a fluctuating disease, Figure 2 shows that the prerequisites for the use of this design were fulfilled. The IBS sum scores in the two periods were not significantly different. The detection of *L. plantarum *MF1298 in one faecal sample at the end of the washout period indicates that the washout period was too short in this subject. However, because the amount of *L. plantarum *MF1298 detected was small and the recording of symptoms took place in the last week of the three-week treatment period, the possibility for a carryover effect is negligible. In summary, the crossover design turned out to be appropriate.

## Conclusions

The results from our study contribute to focus on the risks of using strains with probiotic properties without scientific evaluation. Not all strains with *in vitro *demonstrated probiotic properties actually "confer a health benefit on the host", and their use may even be associated with unfavourable effects. *L. plantarum *MF1298 might be an unfavourable strain and this should stimulate basic research on the molecular basis of probiotic properties.

## Competing interests

SCL and PGF have received funding from Nofima AS (former Matforsk AS) through LA and KN which in turn have received funding from Nortura BA (former Gilde BA).

## Authors' contributions

SCL, LA, KN, and PGF wrote the protocol. SCL and PGF enrolled subjects and collected data and SCL imported data. SCL, SL, and PGF performed statistical analyses. SCL and PGF drafted the paper, which was reviewed by the other authors. All authors read and approved the final manuscript.

## Pre-publication history

The pre-publication history for this paper can be accessed here:

http://www.biomedcentral.com/1471-230X/10/16/prepub
